# Suggestions

**Published:** 1872-12

**Authors:** 


### ARTICLE III.

### *Suggestions.*

BY A DENTIST.

Having just finished reading the *American Dental Journal*, I am struck with the *oneness* of object that seems to be the motor power to every one who contributes to its pages, viz:

Filling Teeth. And, indeed, this is the principle subject of a majority of the original articles appearing in all the journals devoted to dental science. It is a prolific theme. Who could not write pages on the subject, giving his own views and experience? and without any benefit to the reader. It is simply impossible to tell a man how to fill a tooth. It matters not how much theory he has, as to the proper point for condensation, whether the center or around the borders of a filling, etc.; one must learn by experience how to fill, and must do it according to his own idea.

One of the finest dentists I ever saw, used Abbey's unannealed foil and hand pressure; I see many of his fillings twenty years old, as bright, solid, and good as the day they were put in. Others must use leaden or steel mallets, and gold annealed in Chicago fires. Every one has his own idea. I want smooth points, and annealed foil; and a friend who fills equally well, uses unannealed, and hand pressure. *We* want automatic pluggers.

My object in writing is to discourage so *much* theorizing upon this subject, if possible; it is becoming a bore. There are many other subjects, equally worthy of as much attention; for instance, breaking off a tooth, leaving the pulp protruding; old cases of alveolar abscess; disease of the antrum, etc.

I will only speak of the first, and will ask, does any one know of a painless and sure way of destroying the nerve? To apply nitric acid, is almost death, and the fumes are very unpleasant. The acid nitrate of mercury is pretty good, but not sufficiently certain; at least I have not found it so. I will close for the present, adding that I want facts and figures, not the theory of inexperienced dentists.

